# Highly Selective Fluorescence Sensor Based on Graphene Quantum Dots for Sulfamethoxazole Determination

**DOI:** 10.3390/ma13112521

**Published:** 2020-06-01

**Authors:** Thi Hoa Le, Hyun Jong Lee, Ji Hyeon Kim, Sang Joon Park

**Affiliations:** Department of Chemical and Biological Engineering, Gachon University, Seongnam 13120, Korea; hoale2907@gmail.com (T.H.L.); hjlee2@gachon.ac.kr (H.J.L.); jihyeon@gachon.ac.kr (J.H.K.)

**Keywords:** fluorescence sensor, silica molecularly imprinted polymer, graphene quantum dots, sulfamethoxazole

## Abstract

In our research, a reliable fluorescence sensor for the detection of sulfamethoxazole (SMZ) was developed. This method relies on graphene quantum dots (GQDs) entrapped in a silica molecularly imprinted polymer (GQDs@SMIP), which was synthesized by the polymerization using GQDs, SMZ, tetraethoxysilane (TEOS) and 3-aminopropyltriethoxysilane (APTES) as fluorescence material, template, cross-linker, and functional monomers, respectively. The GQDs@SMIP was characterized by fluorometry, Fourier-transform infrared spectroscopy, transmission and scanning electron microscopies, X-ray photoelectron spectroscopy, and powder X-ray diffraction. The GQDs@SMIP exhibited a good capacity to absorb SMZ from solution, which resulted in the quenching of the GQD fluorescence intensity. The intensity of GQDs@SMIP decreased linearly with the SMZ concentration in the range of 1 to 100 µM with a correlation coefficient of 0.99537. In addition, the fluorescence responses of GQDs@SMIP to interfering substances were investigated. The results indicated that there was no effect of interfering substances on SMZ detection. Thus, the highly selective GQDs@SMIP fluorescence sensor is an effective and promising device for SMZ detection and analysis.

## 1. Introduction

Sulfamethoxazole (SMZ) is one of the numerous antibiotics belonging to the sulfonamide family. SMZ is used widely as an antibacterial agent in drugs for infectious diseases, animal-derived food, and aquatic environments [1,2]. As SMZ cannot be eliminated effectively through common water or food treatment processes, there is a probability of an excess amount of SMZ entering the human body. After prolonged exposure to low concentrations of antibiotics, the human body is bound to suffer from a resistance reaction [3,4]. This risk renders it necessary to determine the SMZ content in pharmaceutical formulations as well as to test residues of SMZ, which should not exceed the regulatory limit in drinking water or in food originating from animals, such as eggs, milk, and meat [5]. Therefore, various SMZ detection methods including spectrophotometry [6], electrochemical methods [7,8], LC/MS [9], HPLC [10,11], solid-phase extraction [12], and voltammetry [13] have been developed.

Recently, fluorescence sensing has attracted the interest of researchers for chemical and biological applications, because they both overcome some of the drawbacks of the other detection methods and are also rapid, convenient, simple, non-polluting, and highly sensitive [14,15]. Various fluorescence sensors have been prepared based on GQDs, which are optical materials possessing outstanding characteristics such as stable photoluminescence, good water dispersity, chemical stability, good biocompatibility, and low toxicity [16,17]. Nevertheless, these GQD-based fluorescence sensors have a significant drawback of low selectivity [18]. With our experience in developing fluorescence methods, we addressed this limitation by combining GQDs and a highly selective molecularly imprinted polymer (MIP) [19,20,21]. Recognition using MIP is a technology that is used to analyze samples based on a mechanism that is similar to enzyme–substrate action [22,23]. Therefore, with combined MIP technology, the selectivity of a GQDs-based sensor would be improved significantly. In recent years, SMZ fluorescence sensors replied on MIP have been reported [2,24,25]. They have emerged as powerful techniques not only because of their strong selectivity but also due to their simplicity, easy-preparation and inexpensive cost, when compared with other SMZ sensing methods which require expensive instruments and complex operating processes [26,27,28,29].

**Scheme 1:**
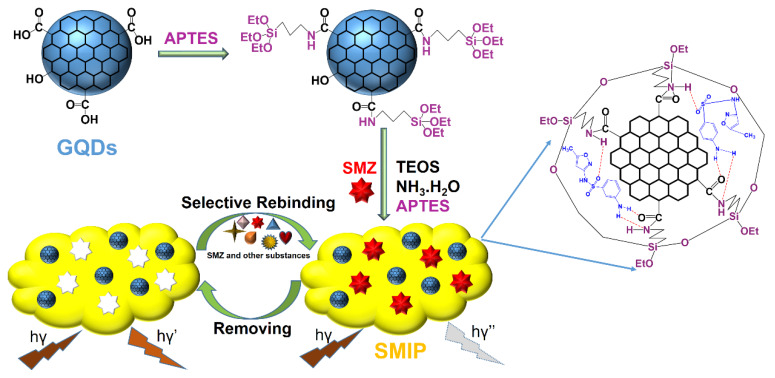
Demonstration of the synthesis and application of GQDs coated with silica molecularly imprinted polymer in sulfamethoxazole detection.

In particular, we synthesized GQDs coated with a silica MIP (denoted as GQDs@SMIP). First, GQDs were prepared from citric acid monohydrate by a thermal process. Subsequently, the GQD surface was modified with APTES, a precursor for silica layer growth, to form GQD-APTES. Next, GQDs@SMIP was fabricated via the sol-gel polymerization of the GQD-APTES complex (as the fluorescent material), SMZ (as the template), APTES (as the functional monomer), TEOS (as the crosslinker), and ammoniacal solution (as the catalyst). SMZ was imprinted on GQDs@SMIP through a non-covalent attachment and could be easily removed by a washing process. After the removal of SMZ, cavities with a shape similar to that of the SMZ structure were left in the polymer, which could recognize and rebind SMZ molecules. Thus, the obtained GQDs@SMIP could serve as a novel material for SMZ detection. Scheme 1 clearly demonstrates the preparation method and application in SMZ sensing of GQDs@SMIP. In this work, we introduced the SMZ analysis in phosphate buffer saline (PBS) by employing GQDs@SMIP. Presently, we are continuing to develop this method for analyzing the various real samples such as human serum, blood sample, drinking water, and food originated from animals.

## 2. Experimental Details

### 2.1. Materials

All chemical substances were obtained from Sigma–Aldrich (St. Louis, MO, USA) and used without further purification. The chemicals include citric acid monohydrate (CA) (CAS: 5949-29-1), NaOH (CAS: 1310-73-2), ethanol (CAS: 64-17-5), APTES (CAS: 919-30-2), TEOS (CAS: 78-10-4), NH_4_F (CAS: 12125-01-8), NH_4_OH (CAS: 1336-21-6), SMZ (CAS: 723-46-6), sulfadiazine (CAS: 63-35-9), sulfamerazine (CAS: 127-79-7), sulfamethazine (CAS: 57-68-1), sulfasalazine (CAS: 599-79-1), sulfapyridine (CAS: 144-83-2), and phosphate buffer saline (PBS) (pH = 9). Deionized (DI) water (pH = 7) (CAS: 7732-18-5) was utilized in all the experiments.

### 2.2. Instruments and Measurements

Photoluminescence (PL) spectra were recorded using a QuantaMaster TM 50 PTI spectrofluorometer from Photon Technology International (Kyoto, Japan). Transmission and Scanning electron microscopy (TEM and SEM) images were collected using FEI microscope (Tecnai, F30S-Twin, Hillsboro, OR, USA) and a field-emission scanning electron microscope (Hitachi S-4700, Hitachinaka, Ibaraki Prefecture, Japan), respectively. Fourier-transform infrared (FTIR) spectra were recorded using a Nicolet 6700 spectrometer from Thermo Scientific (Waltham, MA, USA). Powder X-ray diffraction (XRD) patterns of GQDs@SMIP were recorded on an automated Rigaku D/max 2200 X-ray diffractometer with monochromatic Cu *Kα* radiation (Tokyo, Japan). X-ray photoelectron spectroscopy (XPS) was measured by an X-ray photoelectron spectrometer (PHI 5000, Chigasaki, Kanagawa Prefecture, Japan). The zeta potential was performed on electrophoretic light scattering (Photal Otsuka Electronics, ELS 8000, Osaka, Japan).

### 2.3. Synthesis of GQDs and APTES-Modified GQDs

First, 2 g of CA was taken in a small beaker and heated to 200 °C in an oven. The CA melted, yielding an orange liquid after 30 min. Next, the resulting liquid was dropped into a solution of NaOH (10 mg/mL; 100 mL) and stirred vigorously for 3 h. Finally, the obtained GQD sample was dialyzed using a dialysis bag (MCWO: 1000 Da) for 72 h to obtain pure GQDs.

To synthesize APTES-modified GQDs, 1.5 mL of APTES was dropped to 20 mL of pure GQDs solution (6.85 mg/mL in water solvent). The solution was stirred at 40 °C for 30 min and then cooled to room temperature. The prepared APTES-modified GQDs were purified using petroleum ether, then dispersed in ethanol.

### 2.4. Synthesis of GQDs@SMIP and silica non-molecularly imprinted polymer (GQDs@SNIP)

GQDs@SMIP was formed through a sol–gel polymerization method. First, adding 25 mg of SMZ to 5 mL of ethanol under stirring. Subsequently, 12 mL of the suspension of APTES-modified GQDs in ethanol (volume ratio is 1:1), 2.4 mL of TEOS, and 3.6 mL of APTES were added to the mixture. Next, adding 0.6 mL of a catalytic solution prepared from 2 g of NH_4_F, 23 mL of NH_4_OH, and 100 mL of H_2_O. The mixture was stirred at 25 °C for 30 min. Then, the mixture was centrifuged to recover the product, which was washed with anhydrous ethanol. The SMZ template in the composites was eliminated by washing with methanol until no absorbance peak of SMZ in the washing solvent was obtained. Next, the composites were dried in an oven at 50 °C.

GQDs@SNIP was synthesized in the same manner without adding the SMZ template.

### 2.5. SMZ detection

First, adding 0.1 g of GQDs@SMIP to 100 mL of PBS (pH = 9) under vigorously stirring to prepare a stock solution. Next, a series of 5 mL volume of the stock solution and variety amounts of an SMZ solution were serially dropped into cuvettes. Various mixtures with a final concentration of SMZ of 0, 1, 3, 5, 10, 20, 30, 40, 50, 60, 80, 100, 120, and 140 µM were obtained. The solutions were shaken and then incubated at room temperature for 90 min before fluorescence measurements.

### 2.6. Selectivity

To determine the selectivity of GQDs@SMIP to SMZ, the effect and interferences of other substances belonging to the sulfonamide family such as sulfadiazine, sulfamerazine, sulfamethazine, sulfasalazine and sulfapyridine were investigated.

## 3. Results and Discussion

### 3.1. Characterization of GQDs@SMIP

**Figure 1:**
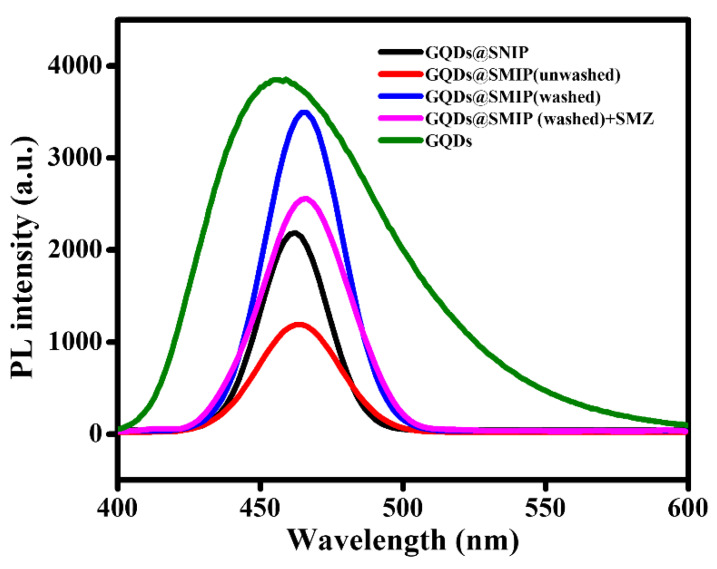
Fluorescence spectra of a GQD suspension (6.85 mg/mL in water solvent), GQDs@SNIP, GQDs@SMIP (unwashed), GQDs@SMIP (washed), washed GQDs@SMIP in the presence of SMZ at 50 µM concentration under the excitation wavelength of 370 nm. The concentrations of GQDs@SNIP and GQDs@SMIP were the same at 5 mg/mL in water solvent.

Fluorescence spectroscopy was applied for characterizing the optical properties of GQDs@SMIP. The fluorescence spectra (Figure 1) shows the maximum emission peak of GQDs is placed at 455 nm, whereas the fluorescence emission maxima of the GQDs@SNIP and GQDs@SMIP red-shifted to 462 and 464 nm, respectively, under the excitation wavelength of 370 nm. This can be explained based on charged Si‒O groups on the surfaces of GQDs@SNIP and GQDs@SMIP. These groups might produce an electric field, which causes the recombination of trapped charged carriers and dangling bonds at the particle surfaces. This led to a change in the position of the emission peak [30,31,32].

Moreover, Figure 1 shows that GQDs@SMIP can be a good material for SMZ sensing. After the SMZ elimination, the fluorescence intensity of GQDs@SMIP increased sharply and when it was recombined with SMZ, the intensity decreased significantly. This is a fundamental characteristic for selective and effective SMZ detection.

**Figure 2:**
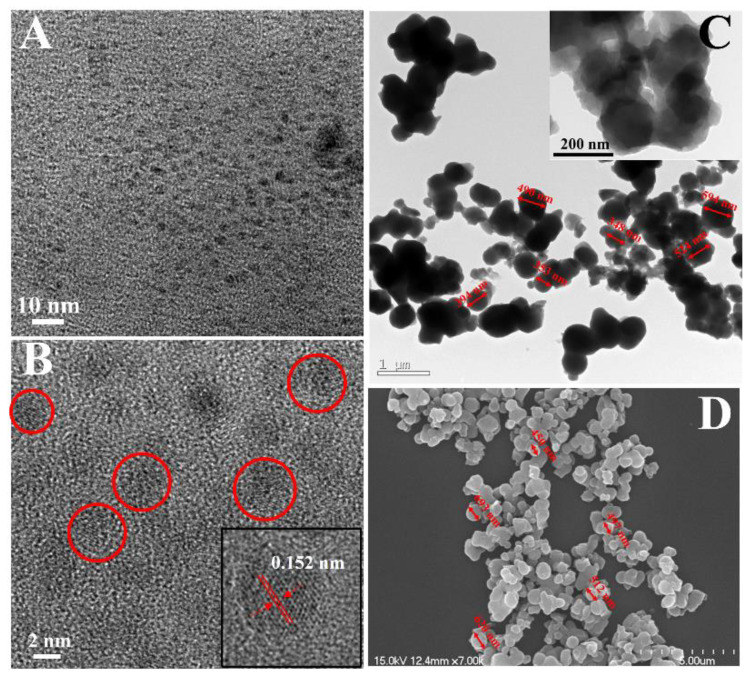
(**A**,**B**) TEM images of GQDs (inset: HRTEM image of GQDs), (**C**) TEM images and (**D**) SEM image of GQDs@SMIP.

The TEM and SEM images in Figure 2 show the morphologies and size of the GQDs and GQDs@SMIP. Figure 2A reveals that GQDs were mono-dispersedly synthesized with a narrow size distribution of 2.7 to 5 nm and an average size of 4.1 nm. In the high-resolution (HR) TEM image (inset of Figure 2B), the crystal structure of the GQDs is apparent, with a lattice parameter of 0.152 nm, which is associated with the graphitic diffraction planes [33,34]. The TEM image of GQDs@SMIP (Figure 2C) displays that the average diameter of GQDs@SMIP is 459 nm, whereas the SEM image (Figure 2D) indicates the average diameter to be 522 nm. Therefore, there is quite good agreement between the TEM and SEM outcomes. Moreover, these results indicate that SMIP might contain many small particles of GQDs.

**Figure 3:**
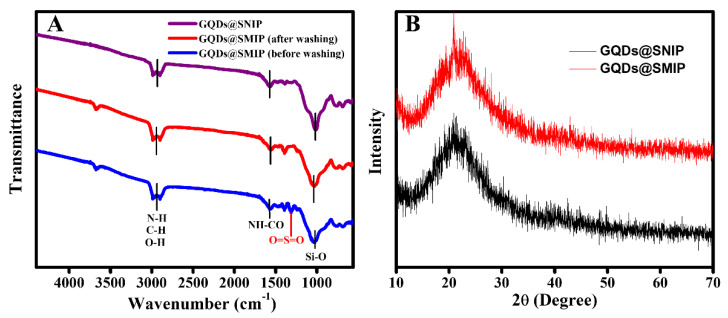
FTIR spectra of GQDs@SNIP, GQDs@SMIP (after washing), and GQDs@SMIP (before washing) (**A**). XRD spectra of GQDs@SNIP and GQDs@SMIP (**B**).

We further characterized GQDs@SNIP and GQDs@SMIP by FTIR spectroscopy. In Figure 3A, the spectrum of GQDs@SMIP (before washing) presents main characteristic peak in the range of 3050–2800 cm^−1^, which may be assigned to the C‒H, O‒H stretching of the carboxylic acid and hydroxyl groups, or N‒H stretching belonging to amine groups. Further, a peak positioned at 1560 cm^−1^, which occurs owing to the HN‒CO bonding vibrations; a peak located at 1297 cm^−1^ results from the vibration of the O=S=O group, which is a part of the SMZ molecule, and a peak at 1017 cm^-1^ occurs due to the stretching vibrations of the Si‒O group. The FTIR spectrum of GQDs@SMIP (after washing) contains all the peaks belonging to GQDs@SMIP (before washing) except for the peak of the O=S=O group, which confirms that SMZ molecules were completely extracted out of the GQDs@SMIP composite. Therefore, after the removal of SMZ, the spectrum of GQDs@SMIP (washed) is extremely similar to that of GQDs@SNIP.

The XRD patterns (Figure 3B) of both GQDs@SNIP and GQDs@SMIP only show a broad peak at ~21°, which could be attributed to the amorphous silica phase (JCPDS No. 29-0085). This result indicates the presence of silica polymer layer in the nanocomposites. Although the TEM image shows a crystalline structure of GQDs (Figure 2B), their size is significantly small as compared to the size of the silica layer; therefore, no specific peak of GQDs was observed in the XRD pattern.

**Figure 4:**
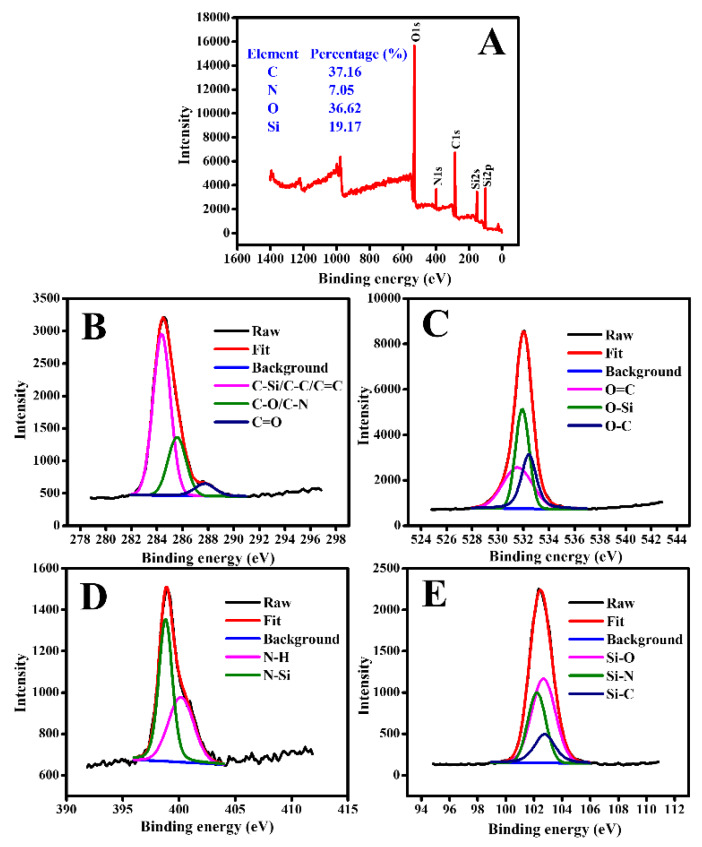
XPS profiles of GQDs@SMIP: survey spectrum (**A**) and high-resolution spectra of C1s (**B**), O1s (**C**), N1s (**D**), and Si2p (**E**).

The XPS profile of GQDs@SMIP (Figure 4A) presents five typical peaks at 529, 397, 282, 150, and 106 eV, correlating with O1s, N1s, C1s, Si2s, and Si2p, respectively. The weight percentages of C, N, O, and Si were analyzed to be 37.16%, 7.05%, 36.62%, and 19.17%, respectively. In Figure 4B, the C1s peak can be de-convoluted into three peaks located at 284.4, 285.6, and 287.8, corresponding to C‒C/C=C, C‒O/C‒N/C‒Si, and C=O bonds, respectively, which originate from the sp^2^ graphitic structure [35] and several carboxyl, hydroxyl, amine, and Si-containing groups belonging to GQDs@SMIP which were further confirmed by O1s, N1p, and Si2p spectra. The O1s spectrum (Figure 4C) was de-convoluted into three characteristic peaks including those of O‒C at 532.4 eV, O‒Si at 532 eV, and O=C at 531.5 eV. The N1s spectrum (Figure 4D) was de-convoluted into two specific peaks corresponding to N‒H at 400.2 eV and N‒Si at 398.8 eV. The Si2p spectrum (Figure 4E) was de-convoluted into a peak at 102.2 eV assigned to the Si‒N bond, and peaks located at 102.7 eV were ascribed to Si‒O and Si‒C bonds.

### 3.2. SMZ Sensing

**Figure 5:**
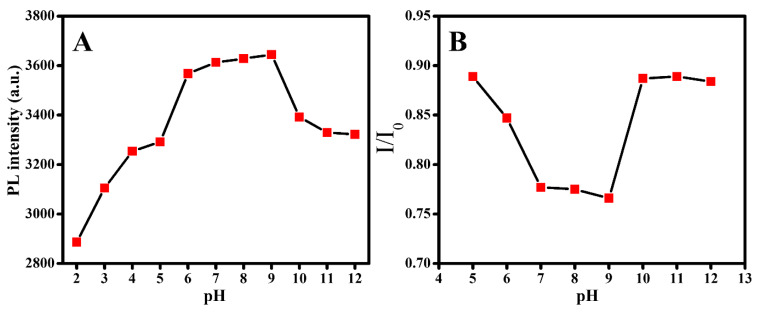
Fluorescence intensity of a GQDs@SMIP suspension (5 mg/mL in water) (**A**) and the quenching effect of SMZ on the fluorescence of GQDs@SMIP (**B**) at various pH values.

We investigated the effect of pH, which is a vital parameter which influences the fluorescence stability of GQDs@SMIP and the fluorescence response of GQDs@SMIP to SMZ. Figure 5A indicates that the fluorescence intensity of GQDs@SMIP is low in strongly acidic or basic solutions. In the pH range of 6 to 9, the intensity reached the maximum value and remained stable. In the presence of SMZ (Figure 5B), the intensity of GQDs@SMIP was strongly quenched at pH values between 7 and 9. It is well-established that if the pH is excessively low or high, the imprinted silica shell can hydrolyze and the template cavities can be destroyed [2,36,37]; this affects the stability as well as the ability of the GQDs@SMIP template to recognize the target analyze. Based on these results, we realized that a pH in the range of 7 to 9 is suitable for SMZ detection. In particular, the pH of 9 is optimum for the investigation of SMZ sensing.

**Figure 6:**
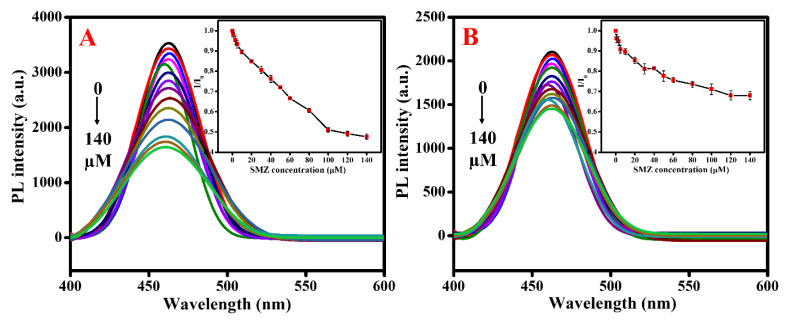
Fluorescence spectra of GQDs@SMIP (**A**) and GQDs@SNIP (**B**) in the presence of different concentrations of SMZ. The concentration of GQDs@SMIP and GQDs@SNIP is 5 mg/mL. Inset: calibration curve for the relationship between *I*/*I*_0_ and the concentration of SMZ.

The fluorescence intensities of GQDs@SNIP and GQDs@SMIP were quenched in the presence of SMZ (Figure 6). This occurred because of hydrogen bonding interaction between SMZ molecules and the amine groups acting as binding sites on the surfaces of GQDs@SNIP and GQDs@SMIP [18,30]. However, there was a key difference in the quenching responses of GQDs@SNIP and GQDs@SMIP to SMZ. Clearly, the quenching degree of GQDs@SMIP was more pronounced than that of GQDs@SNIP. Particularly, Figure 6A,B (insets) shows that the GQDs@SMIP fluorescence intensity quenched sharply corresponding to increasing in SMZ amount from 0 to 140 µM, whereas that of GQDs@SNIP decreased insignificantly. More precisely, in the existence of 140 µM SMZ, the intensities of GQDs@SMIP and GQDs@SNIP at the same concentration of 5 mg/mL were quenched by 53% and 33%, respectively. The presence of specific imprinted cavities in GQDs@SMIP to recognize SMZ led to stronger adsorption of the SMZ template to it. Therefore, SMZ molecules were observed to bind to GQDs@SMIP more strongly, which caused a strong fluorescence quenching effect. In addition, SMZ is known to be a good hole or electron acceptor [38], and an electron transfer process might occur after the entry of SMZ into the imprinted cavities; this is confirmed by a change in the zeta potential of the GQDs@SMIP suspension from −14.75 to −9.08 mV after the addition of SMZ (140 µM). This process also contributes to the quenching phenomenon. The calibration curve in Figure 6A (inset) clearly demonstrates the quenching effect of SMZ on the GQDs@SMIP fluorescence intensity. The intensity decreased steadily and significantly corresponding to the increase in SMZ concentration up to 100 µM. No notable change occurred with further increase because all imprinted cavities might have been filled with SMZ molecules. In the range of SMZ concentration (C_M_) 1 to 100 µM, a good linear relationship was found with the equation, *I*/*I*_0_ = 1 − 0.00555 C_M_ and a correlation coefficient (R^2^) of 0.99537. The experiment was repeated thrice and the result is expressed as the average ± standard deviation. Moreover, the limit of detection (LOD) was determined to be approximately 1 µM. Table 1 shows the result comparison of our fluorescence method with other currently presented methods. It is clear to see that our simple fluorescence approach gives the similar results to that of the others which require expensive instruments and complicated operating process. This is a remarkable advantage of our fluorescence technique using GQDs@SMIP for SMZ detection.

### 3.3. Selectivity

**Figure 7:**
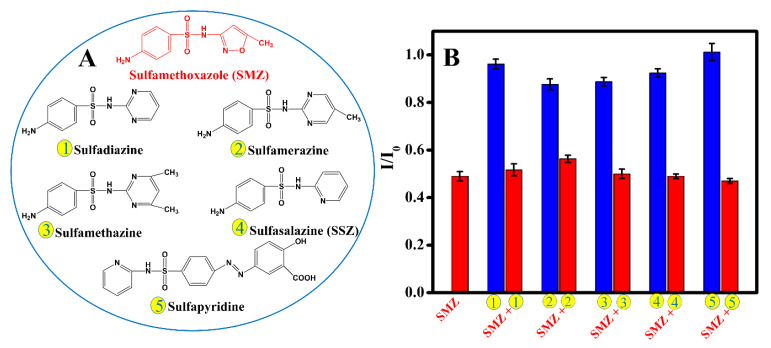
Chemical structures of other analogs belonging to the sulfonamide family (**A**). Responses of GQDs@SMIP to different analogs at the same concentration of 140 µM (**B**).

To verify the specificity of GQDs@SMIP to SMZ, the fluorescent responses of the composite in the presence of five potentially interfering structural analogs belonging to the sulfonamide group were investigated. The chemical structures of sulfadiazine, sulfamerazine, sulfamethazine, sulfasalazine, and sulfapyridine are shown in Figure 7A. GQDs@SMIP contains imprinted cavities, which are specific in shape and size to absorb the SMZ used as the template for imprinting; as other analogues could not be embedded in these cavities, they did not produce remarkable variations in the fluorescence intensity of GQDs@SMIP. However, further adding of SMZ makes the fluorescence of the system decreased significantly (Figure 7B). It may be inferred that the GQDs@SMIP exhibits excellent selectivity in SMZ detection. The experiment was repeated thrice and the result is expressed as the average ± standard deviation.

## 4. Conclusions

We produced GQDs@SMIP via a sol-gel polymerization method. Characterization using fluorometry, TEM, SEM, FTIR, XRD and XPS confirmed that the synthesis approach was successful. Moreover, applying SMZ as a template for the imprinting process resulted in specific absorption cavities for SMZ in the polymeric layer. This imprinting technique combined with fluorescence detection aided us in developing a sensor that is not only effective in SMZ detection, but also has enhanced sensitivity and selectivity. Particularly, the obtained results show that the fluorescence of GQDs@SMIP was quenched with an increase in SMZ concentration over a wide range with a low LOD. This quenching response was not clearly observed with other analogs belonging to the sulfonamide group. With these remarkable features, the GQDs@SMIP sensor can potentially be applied in biomedical systems and environments.

## Figures and Tables

**Table 1:** Comparison of SMZ detection for different methods.

Method	Linear Range	LOD	Reference
**Electrochemical**	0.2–1.4 µM	0.05 µM	[39]
**Electrochemical**	0.8–170 µM	0.8 µM	[40]
**Electrochemical**	2.4–23.6 µM	0.81 µM	[26]
**Electrochemical**	10–100 µM	0.144 µM	[27]
**Ultrasound**	0.78–19.74 µM	0.23 µM	[41]
**Voltammetry**	0.05–70 µM	0.01 µM	[42]
**Our method**	1–100 µM	1 µM	
